# Recent Efforts in Understanding and Improving the Nonideal Behaviors of Organic Field‐Effect Transistors

**DOI:** 10.1002/advs.201900375

**Published:** 2019-08-29

**Authors:** Hio‐Ieng Un, Jie‐Yu Wang, Jian Pei

**Affiliations:** ^1^ Beijing National Laboratory for Molecular Sciences (BNLMS) Key Laboratory of Bioorganic Chemistry and Molecular Engineering of Ministry of Education Key Laboratory of Polymer Chemistry and Physics of Ministry of Education Center of Soft Matter Science and Engineering College of Chemistry and Molecular Engineering Peking University Beijing 100871 China

**Keywords:** current–voltage characteristics, gate voltage‐dependent mobility, nonideal behaviors, organic field‐effect transistors

## Abstract

Over the past three decades, the mobility of organic field‐effect transistors (OFETs) has been improved from 10^−5^ up to over 10 cm^2^ V^−1^ s^−1^, which reaches or has already satisfied the requirements of demanding applications. However, pronounced nonideal behaviors in current–voltage characteristics are commonly observed, which indicates that the reported mobilities may not truly reflect the device properties. Herein, a comprehensive understanding of the origins of several observed nonidealities (downward, upward, double‐slope, superlinear, and humped transfer characteristics) is summarized, and how to extract comparatively reliable mobilities from nonideal behaviors in OFETs is discussed. Combining an overview of the ideal and state‐of‐the‐art OFETs, considerable possible approaches are also provided for future OFETs.

## Graphical abstract

**Nonideal current–voltage characteristics** make the mobility that truly reflects the device performance hard to be extracted. A fundamental understanding is necessary to overcome this issue. Herein, the observed nonideal behaviors of organic field‐effect transistors are summarized and analyzed, and solutions for future device engineering toward ideal organic field‐effect transistors are provided.

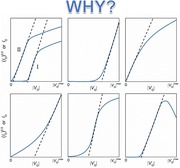

## 1 Introduction

First descriptions of field effect on organic semiconductors trace back to 1970,^1^, ^2^, ^3^ and was first identified by Koezuka et al.^4^, ^5^ in 1987, by using polythiophene as active layer. Over the past 3 decades, unprecedented efforts in the development of organic field‐effect transistors (OFETs) are driven by their potential applications in a wide range of large‐area, low‐cost, solution‐processable, flexible, and stretchable electronics.^6^, ^7^, ^8^, ^9^, ^10^, ^11^, ^12^, ^13^, ^14^, ^15^ Thanks to the improved understanding of structure–property relationship^16^, ^17^, ^18^, ^19^ and charge transport physics,^20^, ^21^, ^22^, ^23^, ^24^, ^25^, ^26^, ^27^, ^28^ impressively high‐performance OFETs have been reported.^29^, ^30^, ^31^, ^32^, ^33^, ^34^, ^35^, ^36^, ^37^, ^38^, ^39^, ^40^, ^41^, ^42^, ^43^, ^44^, ^45^, ^46^, ^47^, ^48^, ^49^, ^50^, ^51^ Field‐effect mobility, μ, has been improved by six orders of magnitude, from low values of 10^−5^ cm^2^ V^−1^ s^−1^ 4, 5 to high values of over 10 cm^2^ V^−1^ s^−1^ 29, 30, 31, 32, 33, 34, 35, 36, 37, 38, 39, 40, 41, 42, 43 that exceed those of thin‐film amorphous silicon‐based transistors (0.5–1 cm^2^ V^−1^ s^−1^). At present, significant progress of OFETs have been made in e‐paper displays,^52^ chemical and biological sensors,^7^, ^8^, ^9^, ^10^, ^12^ simple integrated circuits,^8^, ^11^, ^52^, ^53^ and flexible organic light‐emitting diode (OLED) displays.^52^, ^54^, ^55^

In addition to its own applications, OFET is routinely employed as a test structure for assessing the mobilities of organic semiconductors. The field‐effect mobility of OFET is usually derived from the current–voltage characteristics with the gradual channel approximation (GCA) as Shockley's model for establishing the current–voltage relationships in conventional FETs.^56^ Although many high‐mobility OFETs have been reported, significant nonideal electrical characteristics, where drain current *I*_D_ (or square root of drain current *I*_D_^1/2^) shows a change in slope as a function of gate voltage *V*_G_ in the linear (or saturation) regime, are often observed.^32^, ^33^, ^34^, ^35^, ^36^, ^37^, ^38^, ^39^, ^40^, ^41^, ^42^, ^43^, ^44^, ^45^, ^46^, ^47^, ^48^, ^49^, ^50^, ^51^, ^57^ It is found that almost 55% of the organic thin‐film transistors (OTFTs) with mobilities ≥1 cm^2^ V^−1^ s^−1^ exhibit nonidealities.^58^ Such nonideal behaviors make the classical Shockley's model become inapplicable to accurate mobility extraction.^15^, ^26^, ^28^, ^58^, ^59^, ^60^ In fundamental aspects, mobility obtained from nonideal characteristics is very likely to be neither a useful device characteristic nor a material property. Such a meaningless and artificial mobility impedes our correct understanding of structure–property relationships in both molecular and device engineering. Additionally, incorrect mobility extraction may set erroneous benchmarks for commercialization because OFETs are now being seriously evaluated for mobility critical applications such as current‐driven flexible OLED displays, radio frequency devices, and simple logic circuits. Not to misguide future academic and industrial directions, an extracted mobility that truly reflects device properties is particularly important and making effort on fundamental understanding is necessary.

In fact, the nonideality has been observed^32^, ^61^ and the importance of correct mobility extraction has been highlighted,^26^ also for as long as a decade. However, the origin of nonideality and how to overcome this issue are still not fully understood. In the field, there are a number of excellent and informative reviews. Before overcoming this issue and when nonideality is still unavoidable even after extensive and careful device optimizations, it is highly recommended to follow the suggested operating procedure proposed by Choi et al.^59^ for the measurement and extraction of mobility; this procedure is being regarded as a guideline for the researches of this field. On the other hand, the correct use of classic model in mobility evaluation was underlined and discussed by Horowitz,^26^ Podzorov,^62^ and Sirringhaus^15^ in detail. The possible origins and solutions of double‐slope (i.e., high slope at low gate voltage and low slope at high gate voltage) current–voltage characteristics were carefully reviewed by Nguyen and co‐workers.^63^ In this review, we focus on the scientific understanding of various nonideal behaviors including downward, upward, double‐slope, superlinear, and humped transfer characteristics. The recent efforts on this issue that have not been covered in other reviews will be discussed here. We also review some of the most recent reported state‐of‐the‐art OFETs, and provide possible approaches to overcoming this issue and achieving future OFETs. Additionally, we discuss how to extract comparatively reliable mobilities from nonideal OFETs when nonideality is unavoidable. To establish background knowledge for better understanding the context, we briefly introduce the basic concepts and assumptions of the standard equations for mobility extraction. It is well known that charge injection and charge transport are two decisive roles in OFETs. The former occurs at the metal–semiconductor interface while the latter occurs at the semiconductor–dielectric interface; we therefore fundamentally understand the various nonidealities in device physics along the lines of the two interfaces in Sections 2 and 3, respectively. The nonidealities associated with the nature of semiconducting layer are provided in Section 4. These three sections cover recent efforts on understanding the nonideal behaviors of OFETs, which has not yet been included in other reviews. Lastly, in Section 5, possible effective approaches to ideal, stable, and high‐performance future OFETs are provided along with an overview of the state‐of‐the‐art OFETs with textbook‐like electrical characteristics.

The current–voltage characteristics of OFETs are influenced by many factors, such as charge injection, dielectric property, carrier density, operation surrounding, and, notably, charge transport taking place at interface rather than in bulk; what we evaluate from transfer characteristics therefore is not the intrinsic carrier mobility of a material, but an apparent mobility of a material in a specific device (also referred to as field‐effect mobility). Extraction of field‐effect mobility is always performed with the GCA model based on some assumptions:^64^, ^65^ (1) The transverse gate electric field is much greater than the longitudinal electric field at any position along the conducting channel; (2) the mobility is independent on charge carrier density; (3) the metal–semiconductor interface is ohmic contact. An FET that satisfies these conditions is considered to be an “ideal” transistor. For ideal OFETs, the source–drain current *I*_D_ varies with the gate voltage *V*_G_ at a given source–drain bias *V*_D_. When |*V*_D_| ≪ |*V*_G_ − *V*_T_|, FETs work in linear regime. The current–voltage relationship and the linear mobility (*µ*_lin_) follows Equations (1) and (2)

(1) $$I_{D,\mathrm{lin}}\;=\;\frac{W}{L}\;\mu_{\mathrm{lin}}C_{i}\left(V_{G}\;-\;V_{T}\right)V_{D}$$

(2) $$\mu_{\mathrm{lin}}\;=\;\frac{\partial I_{D,\mathrm{lin}}}{\partial V_{G}}\;\frac{L}{WC_{i}V_{D}}$$

Here *L* and *W* are channel length and channel width respectively, *C*_i_ is capacitance per unit area, *V*_T_ is threshold voltage, and $\frac{\partial I_{D,\mathrm{lin}}}{\partial V_{G}}$ is transconductance $\left(g_{m}\;=\;\frac{\partial I_{D,\mathrm{lin}}}{\partial V_{G}}\;\right)$. As *V*_D_ increases and reaches the pinch‐off voltage (i.e., |*V*_D_| = |*V*_G_ − *V*_T_| = *V*_pinch − off_), the devices get into a working regime called as pinch‐off status. Once |*V*_D_| > |*V*_G_ − *V*_T_|, transistors work in the saturation regime in which *I*_D,sat_ should no longer depend on *V*_D_, and the current–voltage relationship and the saturation carrier mobility (*µ*_sat_) are as follows

(3) $$I_{D,\mathrm{sat}}\;=\;\frac{W}{2L}\;\mu_{\mathrm{sat}}C_{i}{\left(V_{G}\;-\;V_{T}\right)}^{2}$$

(4) $$\mu_{\mathrm{sat}}\;=\;{\left(\frac{\partial \sqrt{I_{D,\mathrm{sat}}}}{\partial V_{G}}\right)}^{2}\;\frac{2L}{WC_{i}}$$

These four equations reveal the linear essence of the relationship between *I*_D_ (*I*_D_^1/2^) and *V*_G_ in linear (saturation) regime in ideality; reliable apparent mobility can therefore be safely evaluated with these equations only when the transfer characteristics are linear. In nonideal current–voltage characteristics, further analysis should be done and/or mobility extraction should be performed by using another revised and proper models and/or equations.^26^, ^66^, ^67^, ^68^, ^69^ In any case, reporters should avoid to extract the mobility from a very narrow, low, or high gate voltage region of only a few volts.

## 2 Origin of Nonidealities from the Metal–Semiconductor Interface: Contact Resistance

**Figure 1:**
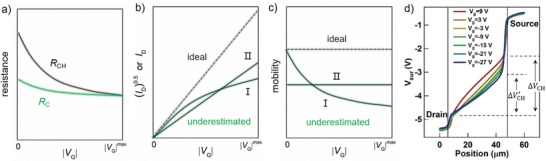
Contact resistance‐induced underestimated mobility. a) Evolutions of *R*_CH_ and *R*_C_ with increasing |*V*_G_|. b) Downward and pulling‐down transfer characteristics due to contact resistance. c) Corresponding evolutions of extracted mobilities as a function of |*V*_G_|. d) Variations of the potential drops across the channel and the metal contact as a function of *V*_G_. Reproduced with permission.^84^ Copyright 2018, IEEE.

Charge injection taking place at the metal–semiconductor interface is the first step for booting up an FET. According to the GCA model, an ideal FET should have ohmic contact at the metal–semiconductor interface; the total resistance (*R*_T_) therefore equals the channel resistance (*R*_CH_), and *I*_D_ should be proportional to *V*_D_ $\left(I_{D}\;=\;\frac{V_{D}}{R_{\mathrm{CH}}(V_{G})}\;\right)$ in linear regime since *R*_CH_ is completely determined by *V*_G_. The extracted linear mobility (above subthreshold region) should be very similar to the saturation mobility, and also the values obtained from four‐probe as well as Hall effect measurements. By contrast, in a practical device, Schottky barrier, which is an electric potential barrier for carriers at metal contact, together with all other factors that hinder charge injection (such as device architecture) is sensitively reflected in contact resistance *R*_C_.^21^, ^28^, ^70^, ^71^ Contact resistance can be derived from 1) unaligned work function;^72^, ^73^, ^74^, ^75^ 2) unfavorable device structure;^26^, ^76^, ^77^, ^78^ 3) unfavorable microstructure of semiconductor nearby the metal contact;^79^, ^80^, ^81^ 4) unfavorable transport properties (mobility and hopping mechanism);^78^, ^82^ and 5) unfavorable dielectric properties.^83^ Total resistance *R*_T_ is the sum of *R*_C_ and *R*_CH_: $R_{T}\;=\;R_{C}\;+\;R_{\mathrm{CH}}\;=\;\frac{V_{D}}{I_{D}}\;$. The contact resistance leads to a potential drop at the metal contact and thus lowers the effective drain bias for driving the charge carriers to pass through the semiconductor; this phenomenon can be directly observed by scanning Kelvin probe microscopy (SKPM) (**Figure** **1**d),^72^, ^84^, ^85^ and the value of *R*_C_ can be evaluated by the Y‐function method, transfer length method, and four‐point probe method.^71^ The effects of contact resistance on current–voltage characteristics have been systematically studied since early 2000, especially by Podzorov,^44^ Horowitz,^26^ and Bao.^86^ Taking into account the contact effect and revising Equation (1) with the actual potential drop across the channel *V*_CH_ = *I*_D_ *R*_CH_ = *V*_D_ − *I*_D_ *R*_C_, Braga and Horowitz provided Equation (5), 26

(5) $$I_{D,\;\mathrm{lin}}\;=\;\frac{V_{D}}{R_{C}\;+\;\frac{1}{\frac{W}{L}\mu_{\mathrm{int},\mathrm{lin}}C_{i}\left(V_{G}\;-\;V_{T}\right)}}$$

where the mobility is intrinsic mobility if the contact issue is the only (or main) source of the deviation from ideality. When *R*_C_ is assumed to be a constant, Equation (5) works well in fitting a downward transfer characteristics (see curve I in Figure 1b for the shape);^26^ this is an initial theoretical description that associates the downward transfer characteristics with *R*_C_. By comparing Equations (1) and (5), the relationship between apparent mobility μ_app,lin_ and intrinsic mobility μ_int,lin_ in linear regime is shown as Equation (6)

(6) $$\mu_{\mathrm{app},\mathrm{lin}}\;=\;\mu_{\mathrm{int},\mathrm{lin}}\;\frac{1}{R_{c}\frac{W}{L}C_{i}\left|V_{G}\;-\;V_{T}\right|\;+\;1}$$

More recently, the corresponding *I*_D,app_ and *I*_D,int_ were associated via Equation (7) by Hu et al.^84^ according to $I_{D,\;\mathrm{app}}\;=\;\frac{V_{D}}{R_{C}\;+\;R_{\mathrm{CH}}}\;$ and $I_{D,\;\mathrm{int}}\;=\;\frac{V_{D}}{R_{\mathrm{CH}}}\;$

(7) $$I_{D,\;\mathrm{app}}\;=\;I_{D,\;\mathrm{int}}\;\left(\frac{R_{\mathrm{CH}}}{R_{\mathrm{CH}}+R_{C}}\right)$$

Equations (6) and (7) reveal that both apparent values of mobility and drain current (μ_app,lin_ and *I*_D,app_) are expected to be underestimated in comparison to their intrinsic values (μ_int,lin_ and *I*_D,int_), as illustrated in Figure 1b,c. Note that sometimes the devices suffering contact issue still exhibit linear character in transfer characteristics; it is because the shape of transfer curves depends on many factors such as carrier density, phonon, and interface scattering, and these effects on the shape of transfer curve can cancel each other out.

Although Equations (6) and (7) sometimes are able to well explain the downward electrical characteristics with underestimated mobility, the drawback of which is not to be able to directly reflect the *V*_G_ dependence on *R*_C_ and *R*_CH_, and also is not to be able to explain all phenomena associated with contact problems such as the double‐slope electrical characteristics. Taking into account the effects of *V*_G_, Bao and Reese^86^ and Liu et al.^67^ developed other expressions for the equations. One type of expressions of the transconductance in linear regime is^67^

(8) $$g_{m,\mathrm{int}}\;=\;\frac{\partial I_{D,\mathrm{lin}}}{\partial V_{G}}\;=\;-\;\frac{V_{D}}{{\left(R_{\mathrm{CH}}+R_{C}\right)}^{2}\;}\left(\frac{\partial R_{\mathrm{CH}}}{\partial V_{G}}+\frac{\partial R_{C}}{\partial V_{G}}\right)$$

This equation points out that *R*_CH_ and *R*_C_ make equivalent contribution to the value of *g*_m_. Equation (8) also reveals that when *R*_C_ is lower and drops down less sensitively with increasing *V*_G_ than *R*_CH_, *R*_C_ becomes the domination of *I*_D_ as *V*_G_ increases (Figure 1a), and thus pulling down *g*_m_ and showing downward transfer characteristics with underestimated mobilities (Figure 1b,c).^43^, ^67^ The above explanation for contact‐issue‐induced underestimated mobility has been experimentally identified by Hu et al.^84^ by using SKPM. Upon the raising of |*V*_G_|, the gradually decreased potential drop across the channel (from Δ*V*_CH_ to $\Delta V_{\mathrm{CH}}^{\prime }$) shows that as |*V*_G_| increased, *R*_C_ was less sensitively decreased than *R*_CH_ and thus the device became more contact‐limited (Figure 1d). After taking into account the effects of *R*_C_, a corrected mobility of 0.26 cm^2^ V^−1^ s^−1^ obtained was higher than the original value (0.11 cm^2^ V^−1^ s^−1^).^84^ We would like to note that such a derivation of Equation (8) explains why Equation (5) well fitted with a downward transfer characteristic only when *R*_C_ was assumed to be a constant;^26^ it is because as *V*_G_ increases the small and *V*_G_‐insensitive *R*_C_ could be approximately considered as a constant in comparison to the large and much more *V*_G_‐sensitive *R*_CH_. Equation (5) can be looked as a special expression associated with Equation (8). Although the expression from of the current–voltage relationship is variable, the natures of those equations are fairly the same in a way.

**Figure 2:**
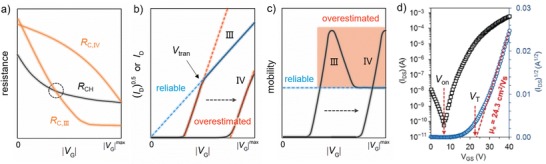
Contact resistance‐induced double‐slope and superlinear nonideal behaviors with overestimated apparent mobilities. a) Evolutions of *R*_CH_, *R*_C,III_, and *R*_C,IV_ with increasing |*V*_G_|. b) Double‐slope transfer characteristics with a “kink” and superlinear transfer characteristics. c) Corresponding evolutions of extracted mobilities as a function of |*V*_G_|. d) Superlinear transfer curve showing a large and extraction‐dependent *V*_T_. Reproduced with permission.^41^ Copyright 2018, WILEY‐VCH.

On the other hand, under two saturation, contact issue can also lead to overestimation of apparent mobility (μ_app_ > μ_int_) in linear region. The first condition is that *R*_C_ is larger than (or comparable to) *R*_CH_ and rapidly decreases to lower than *R*_CH_ with increasing *V*_G_ (see *R*_C,III_ in **Figure** **2**a). In this situation a “kink” appears in transfer characteristics (Figure 2b), extracted from which the evolution of mobility versus gate voltage shows an overestimated peak (Figure 2c). Such an *R*_C_‐induced nonideality with a kink and overestimated mobility is commonly seen in high‐mobility OFETs and has been experimentally demonstrated by Uemura et al.^43^, ^48^ They reported that after improving the contact issue, the transfer curves became linear and exhibited a mobility similar to the value measured by four‐probe measurement. Notably, the ideal transfer characteristics that did not suffer from contact issue overlapped with the double‐slope transfer characteristics at high voltage region; this indicates that in a contact‐induced double‐slope transfer characteristic the mobility extracted in high voltage region with smaller slope is more reliable than the one extracted in low voltage region. In this case, the transition point of gate voltage (*V*_tran_) the kink appears at (Figure 2b) is around the value of *R*_C,III_ that decreases to be very comparable to *R*_CH_ (Figure 2a). Beyond the cross point between the curves of *R*_C,III_ versus |*V*_G_| and *R*_CH_ versus |*V*_G_|, the devices become channel‐limited from contact‐limited. This is the reason why the extracting mobility at gate voltage region is more reliable (even quite accurate).

The second situation also yielding overestimated apparent mobility is when *R*_C_ is larger than *R*_CH_ and *R*_C_ changes less sensitive to *V*_G_ (see curve *R*_CIV_ in Figure 2a).^67^ In this case the transfer curve is superlinear and is with a very large threshold voltage (Figure 2b); as a consequence, the mobility evolution with gate bias is also superlinear (Figure 2c). Actually, the superlinear curve could be regarded as a curve that comes from a shift of the double‐slope curve; it is because only when the gate voltage is high enough the *R*_C,IV_ can decrease down to a comparable value as *R*_CH_. In other words, in the case of superlinear characteristics, the kink of the transfer curve and the peak in mobility may just shift out of the range of measured voltage, so that they were not easily observed. The above discussion is for linear regime at which carrier density is uniform; therefore, extracting mobility from linear regime is judicious in theory.^26^ In saturation regime, by contrast, carrier density gradually changes along the conducting channel (from a maximum near the source contact to practically zero near the drain electrode); the saturation mobility therefore is just a mean value rather than a constant along the channel when the mobility is dependent on the carrier density.^26^ Despite mobility evaluation from saturation regime has a drawback, it is quite often to report a saturation mobility in literatures. The saturated transfer curves also often show nonideality with overestimated mobility. Similar to what we discussed for linear regime, the conventional Equations (3) and (4) are applicable beyond *V*_tran_ and the extracted mobility is more reliable.

For the case of superlinear saturation transfer curve, we would like to make discussion on Figure 2d that is from a recent report on the TFTs of aligned small molecule. Evaluated from this superlinear curve, an electron mobility as high as 24.3 cm^2^ V^−1^ s^−1^ was reported (Figure 2d).^41^ However, the mobility extracted from the linear regime of its output characteristics was just 7.16 cm^2^ V^−1^ s^−1^.^41^ This phenomenon does not meet the character of an ideal FET, and once again, verifying that the apparent mobility extracted from a superlinear transfer characteristic is likely to be a meaningless mobility. Note that *V*_T_ is also a parameter dependent on extraction method, and thus the corresponding extracted *V*_T_ probably does not reflect the true property of the device. At this situation, looking at and comparing the values of *V*_T_ and *V*_on_ may be a good way to check if the very large threshold voltage is artificial (Figure 2d). Similar phenomena are also observed in some other crystal FETs,^87^, ^88^, ^89^ indicating such a type of nonideality is a quite common issue for the FETs of crystals and aligned films. Although detailed resistance analysis was not given in these works,^41^, ^87^, ^88^, ^89^ these observations are consistent with the features of computed results of contact‐limited *I*_D_.^67^ Further exploring the possibility and systematical analysis is needed for better understanding.

**Figure 3:**
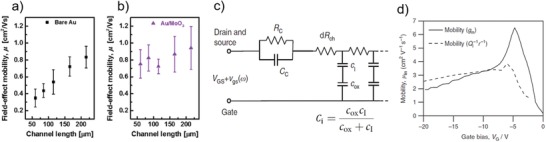
Possible methods for mobility evaluation in nonideal OFETs. Enhancing the channel length: a) Evolution of mobilities with channel length with bare Au. b) Evolution of mobilities with channel length with MoO_3_ modified Au. Reproduced with permission.^73^ Copyright 2016, American Chemical Society. Impedance analysis: c) Equivalent circuit of the transistor impedance. d) Evaluated mobilities as a function of gate bias via from linear transfer curve (solid line, *V*_D_ = −0.1 V) and impedance data (dashed line). Reproduced with permission.^48^ Copyright 2016, Macmillan Publishers Ltd.

Contact issue is a big issue that attracts much attention.^67^, ^72^, ^73^, ^74^, ^75^, ^76^, ^77^, ^78^, ^79^, ^80^, ^83^, ^84^, ^85^, ^87^, ^90^, ^91^, ^92^ It is important to take into account because contact resistance can lead to overestimated and underestimated mobilities. To avoid incorrect mobility extraction, it is undoubted that improving the metal–semiconductor contact is the first choice, and we will discuss the approaches to ohmic contact in detail in Section 5.1. Alternatively, if *R*_C_ is still non‐negligible even after careful device optimizations, a possible way that could be considered is enhancing the channel length. As an example, the TFTs of 6,13‐bis(triisopropylsilylethynyl)pentacene (TIPS‐pentacene) blended with poly[bis(4‐phenyl)(2,4,6‐trimethylphenyl)amine] (PTAA) with bare Au electrodes showed a strong and positive correlation between apparent mobility and channel length (**Figure** **3**a).^73^ After modifying the Au electrodes with MoO_3_ that is a method to improve hole injection, the apparent mobility was much less sensitive to channel length (Figure 3b).^73^ Note that the devices having a long channel length (≈>200 µm) with and without electrode modification also showed average mobilities of around 0.8–0.9 cm^2^ V^−1^ s^−1^. These results reveal that underestimated apparent mobility is more pronounced in small‐size device, and enhancing channel length is helpful to reduce the potential drop across the contact because as long as the channel length is long enough the device is always channel‐limited, maximizing the effective drain bias. A small enough *R*_C_/*R*_T_ ratio enables the classic equations to be applicable in evaluating the intrinsic mobility. However, it should be noted that this way cannot solve the problem fundamentally and may even create new problems because what is desired in practical applications is small‐size devices.

To correctly evaluate mobility, the Y‐function method, four‐point probe and Hall mobility measurements, and revised theory models are highly recommended to be employed. Another approach developed more recently is impedance analysis (Figure 3c).^48^ This method provides access to the channel behavior and mobility at equilibrium condition through Equation (9)

(9) $$\mu \;=\;\frac{1}{Q_{I}r}\;,\;\;Q_{I}\;=\;\int_{\infty }^{V}C_{i}dV_{G}$$

where *r* is sheet resistance and *Q*_I_ is accumulated charge in the channel. The calculated mobility by Equation (9) (dashed line, Figure 3d) reveals that the peak mobility at low voltage region obtained from classic equation is overestimated (solid line, Figure 3d), while their values in the high voltage region are close to each other, which is broadly consistent with what we discussed above.

## 3 Origins from the Semiconductor–Dielectric Interface

**Figure 4:**
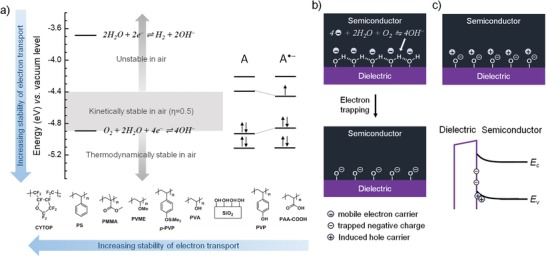
Mechanism of the instability of electron transport. a) Effects of energy levels of organic (semi)conducting materials and chemical properties of dielectrics on the stability of electron transport. b) Diagrams of possible degradation pathway for n‐channel OFETs. c) Diagrams of p‐type doping effects in n‐channel OFETs due to negative trapped charges.

The semiconductor–dielectric interface plays another significant role in device electrical properties because charge transport in OFETs occurs within only a few semiconductor layers near the dielectric. Charge trapping at this interface is well established to cause the undesirable decrease in drain current (sometimes with degradation of mobility), and increase in threshold voltage.^25^, ^93^, ^94^, ^95^, ^96^, ^97^ To improve both the device performance and operational stability, interface engineering.^98^, ^99^, ^100^, ^101^ In comparison to hole transport, stable electron transport in OFETs is much more challenging because electron transport is more sensitive to ambient environment. Although the stability issue of electron transport in OFETs has been studied especially by de Leeuw,^25^, ^95^, ^96^, ^97^ Sirringhaus,^102^, ^103^ Friend,^94^ and other researchers for many years, there is still a need to include a short section about the electron transport and trapping in order to better understand the trapping‐induced nonideal electrical characteristics discussed later.

de Leeuw et al.^22^ first reported that a redox potential of around +0.5 V versus saturated calomel electrode (SCE) (electron affinity equals the redox potential plus 4.4 V) is required for stable electron transport in the presence of water and oxygen (**Figure** **4**a). Electron transport taking place in an energy level approximately deeper than −4.9 eV is thermodynamically stable in air (Figure 4a). Since overpotential (η) is ubiquitous in electrochemical reaction, if η equals 0.5 the energy level for stable electron transport could be up‐shifted to −4.4 eV, but please note that −4.4 eV is a value for kinetically stable electron transport (Figure 4a). While FETs work in n‐channel accumulation mode, negative polanons are created with a formation of in‐gap electronic states, and the location of the in‐gap electronic states in energy is not easy to accurately predict; however, according to experimental data, it can be concluded that a lowest unoccupied molecular orbital (LUMO) level of around −4.0 eV generally meets the requirement for air‐stable electron transport (Figure 4a).^104^ By using spectroscopic technique, Sirringhaus and co‐workers verified that in the trapping process mobile electron carriers became immobile charges and were trapped at OH^−^, via an electrochemical electron transfer between semiconductor and O_2_/H_2_O redox couple^103^

(10) $$O_{2}\;+\;2H_{2}O\;+\;4e^{-}⇌4{\mathrm{OH}}^{-}$$

It has been clear that adsorbed water layer containing solvated oxygen present at the semiconductor–dielectric interface was electron trap sites in real OFETs.^27^

In addition to the requirement related to the nature of semiconducting materials, electron transport was also well established to depend on the dielectric surface. For the most widely used and commercially available dielectric, SiO_2_, the SiOH groups on the surface of SiO_2_ acted as trap sites for electron, and the formation of SiO^−^ is responsible for threshold voltage shift in n‐channel OFETs, according to the following proposed reaction^94^

(11) $$2\mathrm{SiOH}\;+\;2e^{-}\;\;⇌\;\;2{\mathrm{SiO}}^{-}\;+\;H_{2}$$

Reaction (11) reveals that electron carriers can be directly trapped at the interface without the presence of water and oxygen and of course this reaction pathway is reasonable to contribute to the degradation of n‐channel OFETs, but it cannot well explain all phenomena. Therefore, there could be other degradation pathways. A possibility could be an acid‐base neutralization reaction occurring at the interface.^93^ First, as transistors work in the presence of both oxygen and water, the electron carriers are captured by water and oxygen and then trapped at the OH^−^ groups, as shown in reaction (10). Since SiOH is a weak proton acid^16^ and the HO^−^ is a strong base,^15^ acid‐base neutralization reaction therefore probably takes place between them and generates the negative ions SiO^−^ (Figure 4b), as follows^93^

(12) $$4\mathrm{SiOH}\;+\;4{\mathrm{OH}}^{-}⇌4{\mathrm{SiO}}^{-}\;+\;4H_{2}O$$

As a result, the electron trapping in n‐channel OFETs with SiO_2_ gate insulator is possible due to the combination of the strong reducibility of electron carriers and the chemical reactivity of SiOH groups.

After electron trapping occurs, SiO^−^ groups do not contribute to drain current anymore, but contribute to the generation of space charge layer. It should be pointed out that any space charge existing in/on the gate dielectric can lead to a built‐in electric field, which varies the surface potential. The built‐in electric field created by the layer of SiO^−^ groups bends the bands of active layer and induces the injection of hole carriers, leading to a p‐type doping of the semiconductor film near the interface Figure 4c. The existence of the build‐in electric can deplete the negative charge carriers at the interface. Transistor works only when the trapped charges are eliminated and/or the applied gate bias is large enough to overcome the built‐in electric field, therefore resulting in a high threshold voltage.

### 3.1 Trapping of Minority Carrier

**Figure 5:**
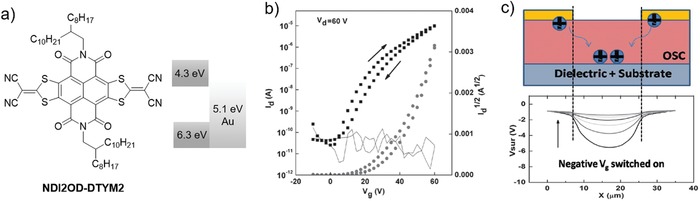
Evidence of injection and transport of minority carrier (hole) in n‐type unipolar OFETs. a) Molecular structure and energy levels of n‐type material NDI2OD‐DTYM2. b) Corresponding saturation transfer characteristics. c) Potential profiles of the channel as a function of time, where the screening effect of potential is indicative of injection and transport of hole. Reproduced with permission.^105^ Copyright 2016, WILEY‐VCH.

In the above discussion, the electrons are the majority carrier in n‐channel transistors; actually electrons can also inject and trap at the interface in p‐channel device where electron is the minority carrier. The injection and transport of minority carrier is commonly observed with low on/off ratio in the devices fabricated with low band‐gap semiconductors. Although the corresponding phenomenon is not observed in unipolar OFETs that exhibit well‐defined OFF‐current across a wide range of gate voltage in transfer characteristics, the injection and transport of minority carriers can indeed occur in unipolar OFETs, which has been verified by Sirringhaus and co‐workers^105^ by using SKPM (**Figure** **5**). Operated in the accumulation mode for electron (depletion mode for hole), the n‐channel NDI2OD‐DTYM2‐based transistor (see Figure 5a for molecular structure and energy levels) showed a negative channel potential initially and followed by a gradual decay in potential; such a gradual screening effect supported the injection and transport of minority carriers (holes) (Figure 5c). The potential decay as a function of time followed an exponential trend of *V*_s_ ∝ exp(−*t*/τ). Evaluated from the potential decay function, the hole mobility of the n‐channel devices of NDI2OD‐DTYM2 was as low as 8 × 10^−9^ cm^2^ V^−1^ s^−1^.^105^

**Figure 6:**
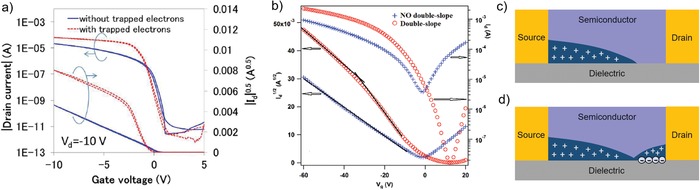
Nonideal electrical characteristics due to trapping of minority carrier (electron) in p‐channel OFETs. a) Nonideal transfer curve with a kink of a p‐type polymer TFT. Reproduced with permission.^105^ Copyright 2015, SPIE. b) Nonideality of transfer curve exhibited in D‐A copolymer PCDTPT‐based FET. Reproduced with permission.^42^ Copyright 2015, WILEY‐VCH. Diagrams of proposed mechanism: c) before electrons trapping and d) after electrons trapping.

When electron is minority carrier, its trapping behavior does not only lead to threshold voltage shift, but also lead to the deviation of p‐channel current–voltage characteristics from linear essence. The minority carrier trapping induced nonideality was first reported by Okachi et al. in 2015 (**Figure** **6**a).^106^ In device of a p‐type polymer with LUMO level of −3.5 eV where device architecture was bottom gate‐bottom contact (BG‐BC) and dielectric layer was self‐assembled monolayer (SAM) modified SiO_2_, when sweeping from positive to negative gate bias, a nonideal double‐slope transfer curve emerged. The slope of the transfer curve at low gate voltage is higher than that at the high gate voltage. Additionally, the slope at the high voltage is very similar to the slope of an ideal transfer curve coming from a same device; this phenomenon suggested that the lower slope at high voltage region is preferable. According to what is discussed at the beginning of Section 3, the electron carriers, which are minority carriers here, are reasonable to inject into channel and be trapped at the polymer–SiO_2_ interface when the gate voltage is positive, leading to the nonideal behavior. Here we would like to differ the nonidealities with a kink due to the contact issue and the trapping of minority carrier. Although the shapes of their transfer characteristics are fairly similar, the mechanisms are different.

This issue was further studied by Nguyen and co‐workers^42^ in an ambipolar FET fabricated with low band‐gap donor‐acceptor (D‐A) polymer poly[4‐(4,4‐dihexadecyl‐4*H*‐cyclopenta[1,2‐b:5,4‐*b*′′]‐dithiophen‐2‐yl)‐*alt*‐[1,2,5]thiadiazolo[3,4‐c]pyridine] (PCDTPT) and with octadecyltrichlorosilane (OTS)‐treated SiO_2_ as dielectric. This device showed a peak of mobility of around 15 cm^2^ V^−1^ s^−1^ at low gate voltage. As a function of stress time, the current of an OTS‐SiO_2_ device decreased while the current of a BCB (a crosslinked polymer dielectric material known to have much less SiOH) device was relatively stable with a quite linear transfer curve. As shown in Figure 6b, the combination of the phenomena including positively shifted turn‐on voltage, depressed electron current, increased hole current, and increased on/off ratio supported that electron trapping occurred in the device of D‐A copolymer. It should be noted that although the SiO_2_ was modified with OTS, the surface probably still had some residual SiOH groups.

The mechanism of nonideality induced by trapped minority carrier was further studied by Okachi et al. very recently.^107^ Since in hole‐accumulation mode the electrons come from drain electrode, thereby these additional electrons mainly trapped nearby the drain contact (Figure 6d). Additional mobile hole carriers induced by the trapped electrons contribute to the drain current, but those additional holes are not taken into account of the carrier density. However, the transconductance always depends on charge carrier density in organic transistors. Therefore the underestimated carrier density leads to an overestimated apparent mobility. Such a phenomenon is just observed at low gate bias region because the negative trapped charges will be depleted as the negative bias goes up. In other words, the mobility extracted from the high bias region is comparatively reliable.

**Figure 7:**
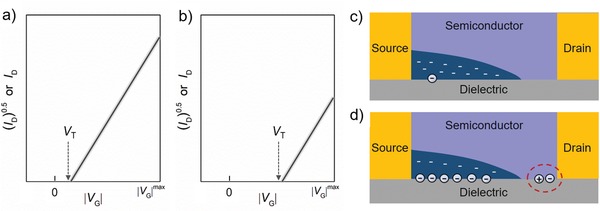
Possible nonideal electrical characteristics due to trapping of minority carrier (hole) in n‐channel OFETs. a) Transfer characteristics of n‐channel OFETs with nearly perfect interface showing a small *V*_T_. b) Transfer characteristics of n‐channel OFETs with unfavorable interface showing a large *V*_T_. c) Diagram of proposed mechanism for (a). Since the interface is nearly perfect, only a very small amount of charges can be trapped. d) Diagram of proposed mechanism for (b). Since the interface is unfavorable for charge transport, electrons are largely trapped while minority carriers (holes) are slight trapped at the interface.

So far, the nonidealities due to trapping of minority carriers have been observed in TFTs operated in hole‐accumulation mode, while holes trapping‐induced nonidealities in TFTs operated in electron‐accumulation mode have not been reported. If the minority carriers are holes, how the injection of holes affects the electrical behaviors of n‐channel devices would be an interesting question. Herein, we would like to discuss the possibility under two conditions: n‐channel transistors with and without a nearly perfect semiconductor–dielectric interface. If the interface of the n‐channel device is nearly perfect; presumably, the trapping of holes is less possible to occur and the transfer curve is quite linear and shows a small *V*_T_ due to a very small amount of trapped negative charge (**Figure** **7**a). It is because for most dielectric materials such as SiO_2_ and poly(4‐vinylphenol) (PVP), they are good electron acceptor and less favorable hole acceptor and thus holes are more difficult to be trapped at the interface than electron, according to the fact that SiO^−^ and PhO^−^ anions are quite chemically stable. Therefore, if the interface is high quality enough to prevent electron trapping, in the negative voltage region, the injected holes probably would not be trapped (Figure 7c). On the other hand, if the interface of the n‐channel device does not undergo good quality modification (such as bare SiO_2_) and has defects and trap sites, the injected holes could be trapped and the additional electrons induced by the trapped holes are much more possible to be trapped at the interface near the drain electrode and do not contribute to charge transport (Figure 7d). When the gate bias raises up to be positive, the electron carriers should be largely trapped at the defective interface, and hence leading to a very large threshold voltage (Figure 7b). We would like to note that the discussion of this part is an open issue; further experimental verification needs to be performed for better understanding.

### 3.2 Semiconductor‐Independent Charge Trapping

The charge trapping discussed above is related to both the essence of organic semiconductors (i.e., the stability of the carriers, the energy levels of the semiconductors, etc.) and the properties of dielectrics. During the trapping process, the original mobile charge carriers are captured from the semiconductor and then become immobile trapped charges; therefore, we refer this class of trapping to as “semiconductor‐dependent charge trapping” here. Actually, there is another trapping process that is less investigated and occurs despite the absence of semiconductor layer. This phenomenon was first reported by Mathijssen and co‐workers^96^ in a semiconductor‐free FET by SKPM. Since the trapped charges in this case are not captured from semiconductor layer, this type of trapping is referred to as “semiconductor‐independent charge trapping.”

**Figure 8:**
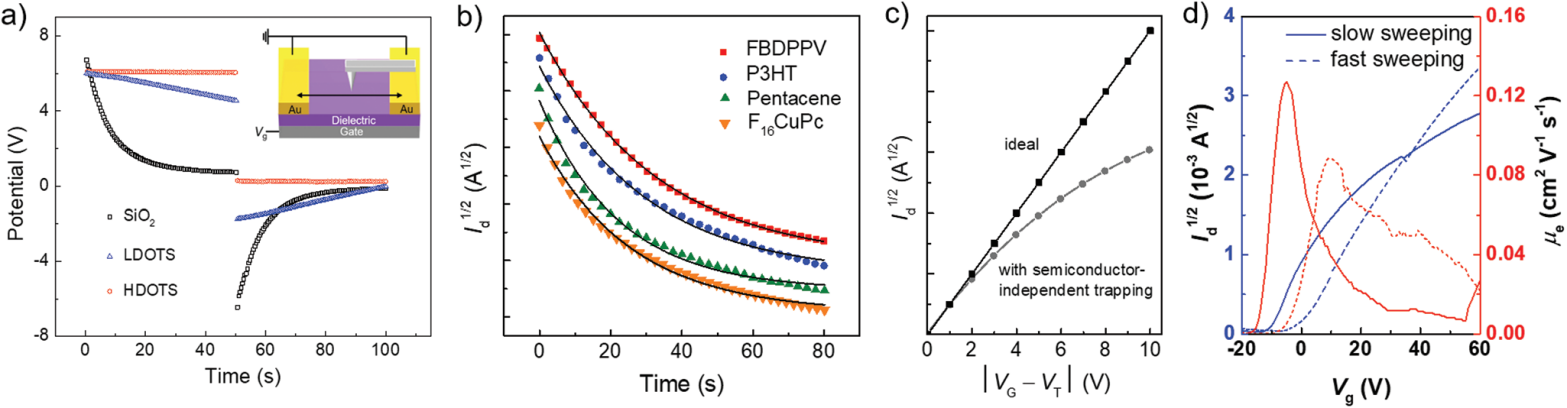
Nonideal downward transfer characteristics induced by semiconductor‐independent charge trapping. a) Characterization of semiconductor‐independent charge trapping in semiconductor‐free FETs with bare SiO_2_, low‐density OTS (LDOTS), and high‐density OTS (HDOTS) by SKPM, where a DC gate bias of +10 V was applied for 50 s first, followed by an application of 0 V for 50 s. The source and the drain electrodes were grounded as a potential reference. b) Experimental evolutions of drain current of the full FETs with SiO_2_ (*V*_g_ = *V*_d_ = −60 V for P3HT and pentacene, and *V*_g_ = *V*_d_ = +60 V for FBDPPV and F_16_CuPc). c) Calculated transfer characteristics with and without semiconductor‐independent charge trapping according to Equation (13), where the increment of effective gate bias is 1 V s^−1^, and assuming τ = 20 s and τ = *∞* for the case with and without trapping. d) Transfer characteristics and apparent mobilities as a function of sweeping rate. Adapted and reproduced with permission.^66^ Copyright 2018, WILEY‐VCH. e,f) Diagrams of proposed mechanism.

More recently we found that semiconductor‐independent charge trapping can occur in various dielectrics, as long as the dielectrics have active functional groups (e.g., Al_2_O_3_, HfO_2_, polyvinyl alcohol (PVA), PVP, poly(allylamine) (PAA‐NH_2_), and poly(acrylic acid) (PAA‐COOH)). In the SKPM experiments performed on a semiconductor‐free FET with bare SiO_2_, the channel potential decayed with time and the decay followed an exponential trend of *V*_s_ ∝ exp (−*t*/τ) (**Figure** **8**a); this trend has been used to describe current decay and threshold voltage shift under bias stress effect. Once we switched the gate bias off, the potential turned into a negative value, indicating that during positive bias stressing, a semiconductor‐independent negative charge trapping at SiOH groups occurred and then gradually screened the applied positive gate bias. Additionally, water was verified to be necessary for this type of trapping.^66^ It should be noted that both positive and negative charge trappings also occurred while negative and positive gate bias was being applied, respectively.^66^

To better understand the relationship between the trapping in the semiconductor‐free devices and the electrical characteristics of the full FETs, Equation (13) for the saturation transfer characteristics was developed by incorporating the potential decay *V*_s_ ∝ exp (−*t*/τ) derived by SKPM into the conventional current–voltage Equation (3)

(13) $$\sqrt{I_{D}}={\left(\frac{W}{2L}C_{i}\mu \right)}^{\frac{1}{2}\;}\;\left|V\prime_{G}\right|\exp\left(-\frac{t}{\tau }\right)$$

where $V\prime_{G}$ is the effective gate voltage $\left(V\prime_{G}\;\;=\;\;V_{G}\;-\;V_{T}\right)$ without the semiconductor‐independent charge trapping, *t* is the time, and τ is the time constant. Equation (13) predicts that at given *V*_G_ and *V*_D_, *I*_D_ would decay exponentially with time, which was experimentally verified in the full TFTs with bare SiO_2_ as shown in Figure 8b. Both p and n‐channel transistors with different semiconductors also decayed with such an exponential trend with similar τ, indicating that the trapping processes here are insensitive to semiconductor and depend on dielectric properties.

Equation (13) reveals that τ and *t* play significant roles in transfer characteristics and predicts that the curve would bend downward when such a trapping occurs (Figure 8c). After passivating the SiOH groups or eliminating the absorbed water at the interface (i.e., equal to increasing the value of τ), the full FETs of poly(3‐hexylthiophene) (P3HT) and fluorinated poly(p‐phenylene vinylene) derivative (FBDPPV) also exhibited nearly ideal transfer curves. Besides τ, the variable *t* revealed that the downward degree would be affected by sweeping rate. The slower the scanning rate we applied, the greater the nonideality behavior became (Figure 8d); since *t* is longer for slow scanning than for fast scanning. Therefore, the transfer characteristics will become more ideal if the sweeping time is largely shorter than the time scale of trapping process (*t* ≪ τ).^66^

The direct connection between the potential decay observed in the semiconductor‐free FETs and the nonideal behaviors of the full FETs via the revised Equation (13) reveals that the semiconductor‐independent charge trapping is a possibility of nonideal behavior of electrical characteristics. This undesired nonideality depends on water, dielectric properties, and sweeping rate, but does not depend on semiconductor, and it generally appears in both p and n‐type polymer. The SKPM results also reveal that SiO_2_ surface is difficult to be completely passivated and the potential can decay as well even after surface modification; this mechanism therefore is a possibility for the downward nonideality that appeared in a large number of high‐performance OFETs where SAM‐modified SiO_2_ was employed as dielectric.^10^, ^11^, ^12^, ^16^, ^18^, ^19^, ^20^, ^21^, ^22^, ^23^

### 3.3 Incorrect Assessment of Gate Dielectrics with Frequency and/or Gate Voltage Dependent Properties

As the mobility of OFETs has been higher than or comparable to that of amorphous silicon‐based TFTs, low operation voltage becomes another increasingly attractive and significant figure of merit for practical applications.^108^ The classic model of OFETs is established via a charge accumulation equation of a simple parallel capacitor: *Q*_m_ = *C*_i_ (*V*_G_ − *V*_T_),^26^, ^28^, ^63^ where *Q*_m_ is the induced mobile charges per unit area; a way to reduce operation voltage therefore is to employ high‐capacitance dielectric materials.^45^, ^46^, ^68^, ^69^, ^109^, ^110^, ^111^, ^112^, ^113^, ^114^, ^115^, ^116^, ^117^, ^118^, ^119^, ^120^, ^121^, ^122^

**Figure 9:**
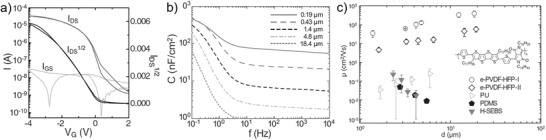
Incorrect mobility extraction due to frequency‐dependent capacitance. a) Transfer curve of PTDPPTFT4 TFT with e‐PVDF‐HFP (*V*_D_ = −15 V). b) Frequency‐dependent capacitance. c) Mobilities of PTDPPTFT4 TFT with different dielectrics (*V*_D_ = −15 V) as a function of the thickness of dielectrics. Reproduced with permission.^118^ Copyright 2016, WILEY‐VCH.

**Figure 10:**
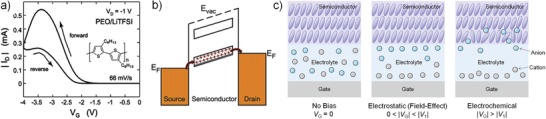
Nonideal transfer characteristics of OFETs using ion‐containing dielectric layer. a) Transfer curve of P3HT TFT (*V*_D_ = −1 V). b) Mechanism for the humped transfer characteristic with negative transconductance. a,b) Reproduced with permission.^109^ Copyright 2007, American Chemical Society. c) As the gate voltage increases to a high enough value at which the anions can start to diffuse into the semiconductor layer, the bulk of the semiconductor layer is doped by the anions (electrochemical doping mechanism).

Notably, however, some of the novel high‐capacitance dielectrics have frequency‐dependent capacitance and/or gate voltage dependent working mode. Calculating the mobility with a capacitance obtained at improper frequency might lead to incorrect assessment. As evidenced by Bao's group,^118^ the capacitance of poly(vinylidene fluoride‐hexafluoropropylene) (e‐PVDF‐HFP) was stable at higher than 10 Hz while sharply increases at lower than 1 Hz (**Figure** **9**b). As the electrical properties of OTFTs are typically acquired by quasi‐static approaches, the capacitance values at the lowest frequency limit are most likely suitable for analyzing the field‐effect mobility. If using capacitance acquired from a normal LCR meter measured at the base frequency of 20 Hz, unexpectedly high mobilities, ranging from 35.1 to 393.8 cm^2^ V^−1^ s^−1^, depending on the thickness of the dielectric layer, were calculated for the (poly(tetrathienoacene‐diketopyrrolopyrrole) (PTDPPTFT4))‐based OTFTs with e‐PVDF‐HFP (Figure 9c). These values are one or two orders of magnitude higher than that obtained in the TFTs with conventional SAM‐modified SiO_2_ for PTDPPTFT4 (0.36 cm^2^ V^−1^ s^−1^).^118^ Such a large extent of increase in mobility cannot be reasonably explained by more effective interfacial trap filling due to higher charge carrier density.^68^, ^116^ The overestimated mobility values are the results from the underestimated charge carrier density carried out by LCR meter at high frequencies (>10 Hz). After correcting the capacitance, the mobility became consistent with that of OTFTs with conventional dielectrics.^118^ Electrolytes are also a class of dielectrics identified to have frequency‐dependent capacitance since their large capacitance values come from the formation of electric double layer (EDLs) that is a process associated with ion migration (**Figure** **10**c).^68^, ^109^, ^111^, ^119^ Some biomass dielectric materials containing ions such as bovine serum albumin^45^ were also reported to be frequency dependent. When novel dielectrics are employed, in particular, result in an unexpected mobility value, careful investigation of their properties is required.

In addition to frequency dependence, for electrolyte‐gated OFETs, voltage dependence is another factor that could lead to deviation from ideality and unreliable mobility assessment. As gate bias goes up to higher than the critical voltage at which the mobile ions can start to permeate into the semiconductor layer (Figure 10c), the devices no longer work with the mode of EDLs, but a mode called as electrochemical doping.^119^, ^121^ In this mechanism, the nature of charge transport becomes bulk transport, rather than the interface transport that is what the traditional model bases on;^68^, ^109^ the classic model of OFETs therefore breaks down here. A simple and quick way to determine the working mechanism is to analyze the output characteristics. Charge carriers accumulating at the interface shows a square‐like output curve with clear pinch‐off voltages and saturation regions whereas bulk transport does not display this classical behavior of output characteristics.^120^ Another phenomenon that may appear along with the electrochemical doping is the humped transfer characteristics with negative transconductance (Figure 10a), which has been observed in single crystal^110^, ^111^, ^113^ and thin‐film^109^, ^112^, ^114^ transistors by Frisbie's group. An explanation for the negative transconductance is the nearly completely filled highest occupied molecular orbital (HOMO) band with holes due to overdoping (overcharging) (Figure 10b) because completely filled or completely emptied band does not contribute to charge transport.^109^ Another possible explanation is that in high voltage region the permeation of ions into semiconductor layer disrupts the original packing of conjugated molecules, as well as leads to scattering effect of charge transport (Figure 10c).^109^ There is a need to point out that the ion‐based transistors working with electrochemical doping mechanism are a class of transistors emerging more recently. Although their working principle is different from the conventional OTFTs, we included this part here because of the fact that ion‐containing dielectrics have been often used for low‐operating voltage OFETs, so that we would like to remind researches to pay attention to the operation condition and capacitance evaluation when using ion‐containing dielectrics. In any case, to correctly understand the electrical properties of a device, taking into account the influences of frequency,^117^, ^118^, ^120^ gate bias,^68^ as well as moisture^45^, ^122^ on dielectric properties is encouraged and even necessary in some cases.

To address the issue of charge carrier density (or capacitance) estimation, several techniques have been developed.^68^, ^69^, ^118^ One is to measure the capacitance as a function of frequency or gate voltage using the AC impedance technique.^69^ Using the capacitance measured at low frequencies (close to DC limit) allows to extract carrier mobility from classical Shockley's model. On the other hand, when capacitance is measured versus gate voltage at low frequencies, charge density can then be obtained by integrating capacitance versus gate voltage following $p\;=\;\int \frac{C_{i}}{edV_{G}\prime }$, where *p* is total charge density (cm^−2^), so that carrier mobility can be expressed as^69^

(14) $$\mu \;=\;\frac{L}{W}\;\cdot \;\frac{I_{D}}{V_{D}ep}$$

The second technique is to measure the gate displacement current upon the application of a gate voltage with zero (grounded) source and drain bias.^69^ The forward sweep and the reverse sweep correspond to charging and discharging a capacitor, respectively. Charge density is determined by integrating the displacement current of the forward sweep with respect to the gate voltage via^69^

(15) $$p\;=\;\frac{Q}{eA}\;=\;\frac{\int I_{G}dV_{G}}{r_{v}eA}$$

where *I*_G_ stands for the gate displacement current (A), *r*_v_ is gate voltage sweep rate (V s^−1^), and *A* is channel area (cm^2^). The displacement current measurement is a quasi‐static DC measurement and is expected to approach the capacitance measurement at low frequencies, but deviates at high‐frequency limit. Both above methods have been successfully applied to determine the charge densities in electrolyte‐gated organic single crystals.^69^

The third technique is to measure the voltage drop across the dielectric layer by using an resistor‐capacitor (RC) circuit at the quasi‐static limit.^118^ The measurement is performed by charging a capacitor with a known resistor, meanwhile the voltage drop on the capacitor is monitored. The voltage drop on the capacitor *U* is described by $\ln\left(1\;-\;\frac{U}{U_{0}}\right)\;=\;-\;\frac{t}{RC}$, where *R* is the resistance of the known resistor, *C* is targeted capacitance at the quasi‐static limit, and *U*_0_ is the target voltage. The capacitance can be calculated via the slopes from the linear fit as a function of *t*. By using this method, the capacitance of e‐PVDF‐HFP is found to be 320–370 nF cm^−2^, which is one order of magnitude larger than the values obtained from LCR meter (at 20 Hz), and is very consistent with the value at low frequency limit. The results show that the measured capacitance depends on the charging time.

These techniques offer alternative approaches to reliably quantify the capacitance and mobility of those devices with frequency‐dependent‐*k* dielectric materials. The conventional measurement procedures can be applied to the dielectric only when the contributions from ion effects are ruled out. It should be noted that although sometimes the transfer characteristics are quite linear, classical model is still not proper to be employed as long as the evaluated capacitance does not reflect the true properties. Here, we would like to emphasize that the issue of novel dielectrics with very high capacitance does not question their advantages, but the properties of this type of dielectrics just need to be carefully verified at specific environment.

## 4 Origins from the Semiconductor Layer

Organic conjugated molecules are bonded together by weak π–π and Van der Waals interactions. Such a type of weak intramolecular interactions results in disordered microstructure. On the other hand, the dipole disorder of dielectric also broadens the density of states of semiconductor. All the chemical and physical defects, and grain boundaries lead to tail states. The lack of extended states and the presence of tail states lead to a gate voltage‐dependent (charge carrier density) mobility. The presence of shallow trap states may cause upward transfer characteristics with a large subthreshold region. Additionally, the complex and inhomogeneous microstructure of organic semiconductors due to the weak intermolecular interaction results in some properties that do not observed in inorganic materials; therefore, the electrical characteristics deviate from ideality and classic charge transport models become inapplicable.

### 4.1 Different Transport Properties between the Bulk and the Surface of Semiconductor

**Figure 11:**
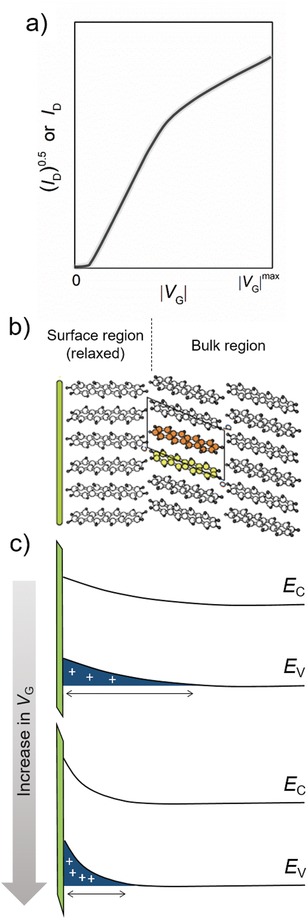
Nonideal electrical characteristics induced by different charge transport properties between the semiconductor bulk and the semiconductor–dielectric interface. a) Diagram for the shape of nonideal current–voltage characteristics. b) Schematic for the different structures of the surface and the bulk of tetracene single crystals derived from the experimental data. Adapted with permission.^123^ Copyright 2014, Macmillan Publishers Ltd. c) Diagrams for the extent of carrier distribution in the direction of the thickness of active semiconductors in OFETs. Top is for weakly gate biased devices and bottom is for strongly gate biased devices.

Different transport properties between the bulk and the surface of the semiconductor layer can lead to nonideal behavior in OFETs. This possibility was first proposed by Takeya et al.^32^ in 2007. They found that in rubrene single crystal FETs the transfer characteristics showed a high slope at low voltage region and small slope at high voltage region. The mobility extracted at low gate voltage region is almost three times higher than that extracted at high voltage region. Although this type of nonideality was identified to be able to result from contact resistance, in this case the contact issue is not the origin because the current–voltage characteristics carried out by four‐probe measurements still exhibited pronounced nonideal behavior. Further investigation experimentally demonstrated that molecular packing mode of the semiconductor layer near the surface can be different from that of the bulk due to their different surrounding. In tetracene single crystals, the first layer of the semiconductor on the substrate‐side suffered a surrounding different from that in the bulk; therefore surface relaxation of microstructure of the crystals occurred (**Figure** **11**b).^123^ The surface was less conductive than the bulk in the tetracene crystals, and their transfer integrals and energy dispersions showed large differences. Density functional theory calculation showed that the bulk and the surface mobilities were 1.2 and 0.3 cm^2^ V^−1^ s^−1^ at 300 K.^123^ Upon an application of low gate bias, the carriers extend into the higher order (higher mobility) bulk, while the greater the gate voltage is, the tighter the charge carriers confine at the conducting interface (Figure 11c); charge carriers therefore might have a lower mobility at high gate voltage region. This explanation is consistent with the result obtained in a high gate voltage FET. Different transport properties between the bulk and the surface of the semiconductor layer are indeed a possibility for nonideality, and the higher disorder degree of the first few semiconductor layers than the bulk of semiconductor leads to nonideal current–voltage characteristics.

### 4.2 Carrier–Carrier Interaction

The electrical characteristics of another rubrene single crystal with highly polarizable Ta_2_O_5_ gate dielectrics also exhibit pronounced deviations from those of conventional FETs, and also show a higher transconductance at low voltage region than at high voltage region.^61^ The temperature‐dependent drain current showed an unusual phenomenon, which is the activation energy *E*_a_ increased with increasing carrier density *n* for the densities larger than 0.02 holes per molecule. Fratini et al.^61^ explained that could be due to the effects of the Coulomb interactions between hole carriers. At those densities, the charge carriers accumulated in the conducting channel of the crystal, and the average distance between the carriers is only a few molecules; therefore the resulting (bare) Coulomb interaction is a few hundreds of millielectronvolts, much larger than the thermal energy at room temperature. The higher the gate bias, the greater the Coulomb interactions, and thus the resulting unusual activation energy evolution leads to the electrical characteristics deviations.^61^

### 4.3 Quasi‐1D Charge Transport

**Figure 12:**
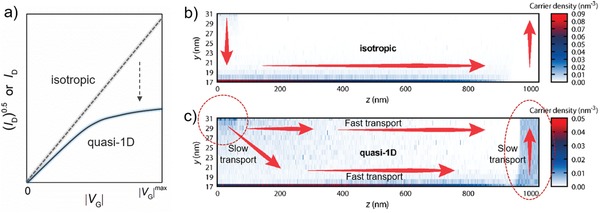
Quasi‐1D transport induced nonideal behavior in OFETs. a) Diagram for stimulated shape of nonideal transfer characteristics with a kink due to strong anisotropic charge transport, where the anisotropy was assumed to come from increase in the thickness of semiconductor layer (increase in *d*) or the reduction of mobility in other transport direction (decrease in *µ*_⊥_). Stimulated charge carrier density distribution in the organic semiconductor layer in the simulated OFET device for b) isotropic and c) quasi‐1D (*µ*_//_/*µ*_⊥_ = 2000) charge transport. Arrows illustrate the flow of charge carriers. Adapted and reproduced with permission.^124^ Copyright 2018, American Chemical Society.

In a recent report, Brédas and Li^124^ proposed that the observed nonideal current characteristics, in particular, in organic crystal or highly oriented polymer FETs, might be due to the quasi‐1D nature of charge transport. In the model of quasi‐1D charge transport, the mobility along the source‐to‐drain direction is much faster than that in the other directions. A fraction of the charges travels through the bulk to the drain electrode without reaching the channel (**Figure** **12**c) due to the highly anisotropic carrier mobility. In their Kinetic Monte Carlo simulations, the transfer characteristics of bottom gate‐top contact (BG‐TC) transistors showed a kink and a higher slope in low voltage region; it is because increasing the gate voltage trends to pull the mobile charge carriers closer to the conducting channel, thus increases the portion of the transport along the other directions and reduces the portion of bulk transport and thus raising up the difficulty in charge injection and collection at the contact (traveling through the semiconductor layer) (Figure 12c). In this quasi‐1D transport model, charge transport in the bulk of semiconductor becomes significant and the GCA model breaks down.

Quasi‐1D charge transport is expected to occur and affect the device physics when the following two conditions are satisfied: (1) the actual charge injection region is far away from the accumulation layer and (2) the semiconductor layer is highly ordered, aligned, and/or oriented (such as organic crystals and highly oriented polymer films) where mobility is highly anisotropic. The farer the distance (increasing the thickness of semiconductor layer) and the higher the level of anisotropy lead to an increased ratio of bulk transport to interface transport, making stronger deviation from the GCA and increasing the degree of nonideality.

The authors differed this mechanism from the mechanism of nonohmic contact and pointed out that the extracted mobility in the low gate voltage region was not overestimated in this case.^124^ However, a number of factors in this case are in line with the mechanism of contact resistance. For example, as the authors noted that quasi‐1D charge transport is also possible to affect the operation of BG‐BC devices. They explained that it is because poor morphologies at the metal–semiconductor interface have been suggested to be able to result in worse carrier injection compared to the case of a BG‐TC device, along with the actual charge injection happening away from the channel. However, poor morphologies at the metal–semiconductor interface in BG‐TC devices have been identified to be a reason for increase in contact resistance.^125^, ^126^, ^127^ Similarly, increasing thickness and lowering out‐of‐plane mobility lead to space charges in the access region near the metal contact,^127^, ^128^ as what the authors show in Figure 12c. The high carrier density near the charge injection and charge collection regions also has been demonstrated to be able to lead to increase in contact resistance. Lastly, we would emphasize that although there are some uncertainties, the proposed mechanism of quasi‐1D charge transport indeed provides one more possibility for the researches' consideration. Moreover, it is reasonable that different mechanisms could simultaneously contribute to the nonideal behavior of OFETs. Additional experimental data for confirming the possibility of anisotropic charge transport and excluding the effects of other factors such as nonohmic contact are needed.

## 5 Solutions

### 5.1 Achieving Ohmic Contact

Contact resistance has always been a big issue and attracted much attention in the development of OFETs,^67^, ^72^, ^73^, ^74^, ^75^, ^76^, ^77^, ^78^, ^79^, ^80^, ^83^, ^84^, ^85^, ^87^, ^88^, ^89^, ^90^, ^91^, ^92^ especially when the size of device is reduced into smaller and smaller because *I*_D_ is no longer limited by *R*_CH_ but rather by *R*_C_. In the following section, a number of possible approaches to ohmic contact are given, along with an overview of the recent progress of achieving ohmic contact.

In reality, *R*_T_ equals the sum of *R*_CH_ and *R*_C_. *R*_C_ mainly consists of two contributions: the interface resistance *R*_int_ and the bulk resistance *R*_bulk_. *R*_T_ therefore is given by the sum of *R*_CH_, *R*_int_, and *R*_bulk_, and thus the relationship between the effective potential drop across the channel $V\prime_{D}$, *R*_int_, and *R*_bulk_ can be expressed as follows

(16) $$R_{T}\;=\;R_{\mathrm{CH}}\;\left(L\right)\;+\;R_{\mathrm{int}}\;+\;R_{\mathrm{bulk}}$$

(17) $$\frac{V\prime_{D}}{V_{D}}\;\;=\;\;\frac{R_{\mathrm{CH}}}{R_{T}}\;\;=\;\;1\;-\;\frac{R_{\mathrm{int}}}{R_{T}}\;-\;\frac{R_{\mathrm{bulk}}}{R_{T}}$$

According to Equation (16), it is clearly that largely reducing *R*_int_ and *R*_bulk_ are able to enable ohmic contact and correct mobility extraction. *R*_int_ is a result related to the properties of the electrode–semiconductor interface, such as the mismatch of energy levels of electrode and semiconductor and the presence of interfacial dipoles. Several effective methods have been developed and widely used to reduce *R*_int_ in device fabrication. For hole transport, these methods include, but are not limited to, modifying the electrode surface with PFBT SAMs, contact doping with F_4_TCNQ, and inserting a thin metal oxide layer. However, by using these methods, only a few studies reported that ohmic contact was achieved, and *R*_C_ was still non‐negligible (e.g., at least higher than 1 kΩ cm) in many cases. After careful analysis, the key factor that has been neglected in many cases could be the effects of *R*_bulk_. *R*_bulk_ is a resistance that reflects the transport properties of injected charges traveling through the semiconductor layer between the electrode–semiconductor interface and the accumulation layer. It relies on several factors: device geometry structure, the morphology and the intrinsic mobility of semiconductor layer near the metal contact, and the thickness of semiconducting layer. *R*_bulk_ contributes more significantly to *R*_C_ in staggered than in co‐plane devices.

**Figure 13:**
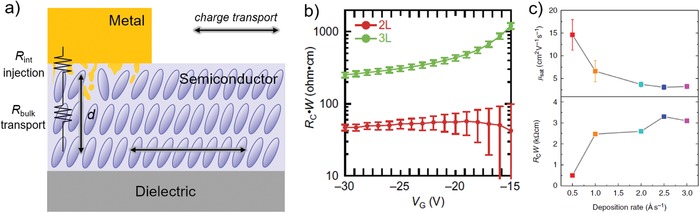
Ideal BG‐TC OFETs with ohmic contact. a) Diagram for BG‐TC device. b) Dependence of the contact resistance on the gate voltage for the two and three layer thick OFETs. Reproduced with permission.^128^ Copyright 2018, AAAS. c) Average field‐effect mobility (top) and width‐normalized contact resistance (bottom) versus deposition rate of metal contact. Reproduced with permission.^125^ Copyright 2018, Macmillan Publishers Ltd.

For staggered devices (e.g., BG‐TC), *R*_bulk_ can be modulated by the thickness and the intrinsic carrier mobility of the semiconductor layer (**Figure** **13**a). A lower thickness of semiconductor layer results in a lower access resistance and a lower density of trap states, and high intrinsic mobility can mitigate the extent of current crowding (i.e., mitigate the space‐charge limitation on injection).^128^ An excellent recent investigation that verified the efficacy of reducing the thickness of semiconductor layer on the decrease in *R*_bulk_ was reported by Takaya and co‐workers.^128^ In the BG‐TC devices they fabricated, the interface of metal contacts was doped by F_4_TCNQ and the thickness of organic single crystals was well controlled to be only two layers. It is not surprised that the former effectively increased the conductivity and reduced the width of the Schottky barrier at the metal contact, and filled the density of trap states in the access region of the crystals, thus largely decreased the *R*_int_. Additionally, together with a significant decrease in *R*_bulk_, which is achieved by using a thickness of semiconductor layer as thin as two layers, a *V*_G_‐independent *R*_C_ down to 46.9 Ω cm was achieved (Figure 13b).^128^ We would also discuss the importance of depositing rate of metal. Recently Lamport et al.^125^ reported that slower depositing rate was able to yield larger grain size of Au. After modified with PFBT SAMs and probed by SKPM, the surface with large grain size showed local maxima in potential while potential of the surface with small grain size was homogenous. They proposed that these local maxima could provide regions of enhanced injection into the semiconductor, leading to a contact resistance (≈200 Ω cm) that is several times lower than the values obtained from high deposited rate of Au (Figure 13c). Such a low contact resistance enables mobilities as high as 19.2 cm^2^ V^−1^ s^−1^ and 10 cm^2^ V^−1^ s^−1^ in the TFTs fabricated with small molecule 2,8‐difluoro‐5,11‐bis(triethylsilylethynyl)anthradithiophene (diF‐TES ADT) and polymer indacenodithiophene‐co‐benzothiadiazole (IDTBT), respectively, notably, achieving linear saturation transfer characteristics.^125^ Additionally, these saturation mobilities were comparable to the values extracted from the linear regime (16 cm^2^ V^−1^ s^−1^ for diF‐TES ADT). These results indicate that slow deposition rate of electrode is an effective method to reduce contact resistance and thus enable reliable high mobility.

**Figure 14:**
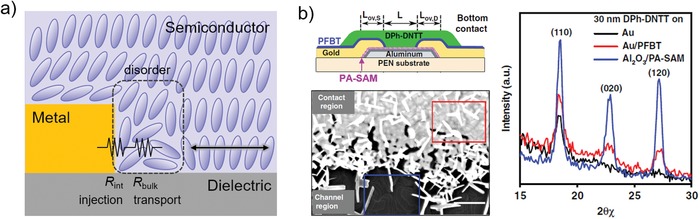
Ideal BG‐BC OFETs with ohmic contact. a) Diagram for BG‐BC device. b) Device structure of a DPh‐DNTT TFT with PFBT‐treated gold contacts and tetradecylphosphonic acid‐passivated Al_2_O_3_ dielectric (Al_2_O_3_/PA‐SAM), and the corresponding SEM and GIXRD image. Reproduced with permission.^127^ Copyright 2019, Macmillan Publishers Ltd.

In comparison to staggered TFTs, co‐planar (e.g., BG‐BC) devices have usually shown or predicted to have higher *R*_C_. It is because of i) the discontinuous coverage and poor film morphology of semiconductor layer near the edge of the contact (**Figure** **14**a),^126^ ii) the absence of metal clusters penetrating into semiconductor layer (Figures 13a and14a),^125^ and iii) the relatively small charge injection area at metal contact.^127^ The first reason leads to decrease in mobility, along with the latter two, resulting in increase in the extent of space charges in the semiconductor, thus increasing the access resistance. The key factor we can modulate for co‐plane structure is to improve the film morphology of semiconductor at the metal contact and optimize the mobility for reducing *R*_C_. In this case, it is less effective to reduce *R*_C_ by tuning the thickness of semiconductor because charge carriers do not need to perpendicularly travel through the semiconductor layer between conducting channel and electrode surface. The main reason resulting in an undesirable film morphology at metal contact is associated with the surface energy. When the surface energies of the metal and the dielectric are similar, good film morphology in the channel region could extend along and across the source and drain contact edges.^126^, ^127^ It has been understood that as the most widely used electrode and dielectric, Au and SiO_2_ have different surface energy. Through treating the Au electrodes with UV/ozone treatment, which induced the formation of a thin AuO*_x_* layer, the AuO*_x_* layer yielded a low surface energy comparable to that of dielectric; therefore the semiconductor molecules tend to align straight on the surface,^126^ which is similar in shape and dimension to those grown on the SiO_2_ gate dielectric in the channel region (edge‐on), decreasing the access resistance of carriers to the channel, which is *R*_bulk_. Additionally, *R*_int_ was reduced due to the decreasing hole‐injection barrier between Au and pentacene because of the insertion of the thin metal oxide. In BG‐BC TFTs of PET, *R*_C_ as low as 80 Ω cm was reported, along with a very weak dependence of mobility on the channel length.^126^ If the dielectric surface is modified with organic SAMs (e.g., Al_2_O_3_/PA‐SAM), organic SAMs (e.g., PFBT SAM) are good candidates of the modification layer for metal electrodes (Figure 14b).^127^ The film morphology of the semiconductor layer on top of the PFBT modified Au electrodes was verified to be similar to the film morphology in conducting channel. Favorable film morphology near the metal–semiconductor interface, ideal injection barrier tuned by the PFBT SAMs, and the intrinsic good transport properties of DPh‐DNTT, *R*_C_ of a few hundreds Ω cm was achieved in BG‐BC devices. By further decreasing the thickness of dielectric layer down to 3 nm, due to a more favorable electric‐field distribution, the contact resistance and its dependence on the gate‐overdrive voltage are reduced overall and a record low *R*_C_ of 29 Ohm cm was achieve on flexible polyethylene naphthalate (PEN) substrates, with *V*_G_‐independent mobility of around 5 cm^2^ V^−1^ s^−1^, low voltage and high frequency application, and a record subthreshold swing of 62 mV per decade.^127^

### 5.2 Use of Bilayer/Nanolaminates Dielectrics

Although using trap‐free materials as dielectrics seems to be a way to avoid trapping‐induced nonideality, mostly commercial available trap‐free dielectrics, such as CYTOP and polystyrene (PS), often have low *k*, thereby relatively low capacitance, high operating voltage, and a broad subthreshold region. Upward transfer characteristics have been quite frequently observed in the OTFTs with low‐*k* dielectric.^129^, ^130^

**Figure 15:**
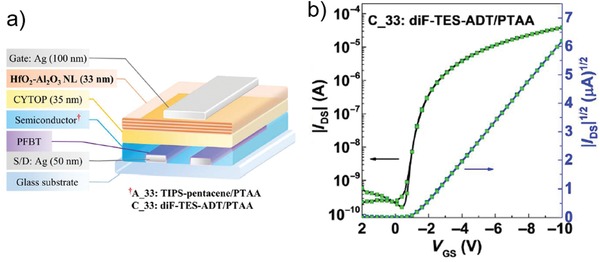
a) Device structure of TG‐BC OTFTs with gate dielectric layers of CYTOP/NLs and b) the corresponding transfer characteristics of as‐fabricated OTFTs. Reproduced with permission.^131^ Copyright 2018, AAAS.

It is evident that the composition of dielectric layer has a high impact not only on the reliability of OFETs, but also on their stability. An approach that has been developed and could meet the requirements of reliable, stable, low‐operating‐voltage, and high‐mobility applications is use of bilayer dielectric or nanolaminates (NLs) (**Figure** **15**b).^131^, ^132^, ^133^ In the bilayer structure, a low‐*k* and trap‐free material (e.g., CYTOP) and a high‐*k* component (e.g., Al_2_O_3_ and HfO_2_) need to be combined. The purpose of the insertion of the low‐*k* layer between the high‐*k* dielectric and the semiconductor layer is to provide a trap‐free interface for charge transport and avoid dipole‐induced energetic disorder of semiconducting layer. The presence of high‐*k* layer ensures low‐voltage operation. Such a bilayer structure (e.g., CYTOP/Al_2_O_3_) combined with electrode modification (if appropriate) provides access to perfect current–voltage characteristics that exhibit linear essence, near zero threshold voltage, and steeper and narrow subthreshold region, along with mobilities higher than or comparable to the values obtained from TFTs with single‐layer dielectrics.^131^ This strategy works well with both p and n‐channel small molecules and polymers.^132^ To further achieve long‐term environmental and operational stabilities, bi‐component NLs such as Al_2_O_3_/ZrO_2_ and Al_2_O_3_/HfO_2_ were developed to instead the high‐*k* dielectrics in the bilayer structure (Figure 15a). Because of the more unreactive and denser nature of NLs, the robust TFTs show outstanding stabilities, even after immersed into 95 °C hot water, the electrical characteristics remain linear.^133^ Measured at room temperature, Δ*V*_T_ under on‐state bias stress (*V*_D_ = *V*_G_ = −10 V) with fitted curves extrapolated to a stress time of over 10 years.^131^ Here, an interesting question could be why the CYTOP/NL structure provides so good stability of *V*_T_ while *V*_T_ of the devices with only CYTOP gradually go higher upon bias stress.^131^, ^134^ A possible explanation is that the NLs appear to compensate for the shift of *V*_T_ induced by the trapping of charge carriers. The presence of NLs produces an opposite *V*_T_ shift over time, for example, via charge accumulation within the dielectrics by slowly oriented dipoles.^132^

### 5.3 Avoiding Ambipolar Charge Transport

**Figure 16:**
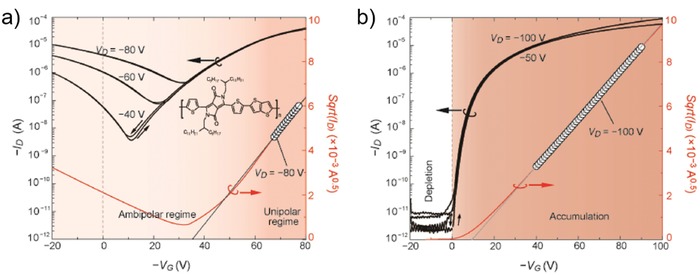
a) Typical transfer characteristics of ambipolar OFETs fabricated with DPPT‐TT. b) Transfer characteristics of unipolar OFETs also fabricated with DPPT‐TT but undergoing contact doping. Reproduced with permission.^135^ Copyright 2016, WILEY‐VCH.

Another possibility that leads to inaccurately mobility evaluation is the ambipolar charge transport behavior in OFETs. Please note that in this section we do not mean the trapping of minority carriers in ambipolar transistors, but the simultaneous hole and electron transport in a device. Since devices operated in linear regime suffer from severe impacts of contact resistance and gate leakage, and for transport analysis and integrated circuit applications, devices working in saturation regime are preferred in some cases. However, high *V*_D_ induces ambipolar charge transports in low band‐gap D‐A copolymers, causing the current–voltage characteristics to be *V*_D_ dependent and deviate significantly from ideality (**Figure** **16**a).^135^ As *V*_G_ increases, the transport behavior transits from ambipolar regime into unipolar regime (Figure 16a). As the commonly used classic equations are developed based on unipolar transistors, and the unipolar regime in an ambipolar transistor always falls within a narrow and high‐voltage range with only a few volts; that means only a small region available for parameter extraction.

A way to extract parameters precisely in low band‐gap D‐A copolymer is to tune the energy levels of the contact and thus block the minority carrier injection. A method potentially useful in this issue is use of contact doping.^135^ For a device where hole is the majority carrier, it has been verified that p‐doped contacts not only enhance the hole injection but also block the electron injection, thus allowing unipolar hole transport and showing ideal hole transport behavior (Figure 16b). The second method is to build an asymmetric charge injection and collection by using asymmetric electrodes,^136^ since hole and electron are extracted and injected from two different electrodes with opposite direction. The third approach is introduction of dipole at the semiconductor–dielectric interface to strengthen the unipolarity of TFTs, such as use of amine‐tailed SAMs for electron accumulation and transport. The dipole due to the presence of amine‐tailed SAMs deplete the accumulation of holes.^137^

## 6 Conclusion and Outlook

**Figure 17:**
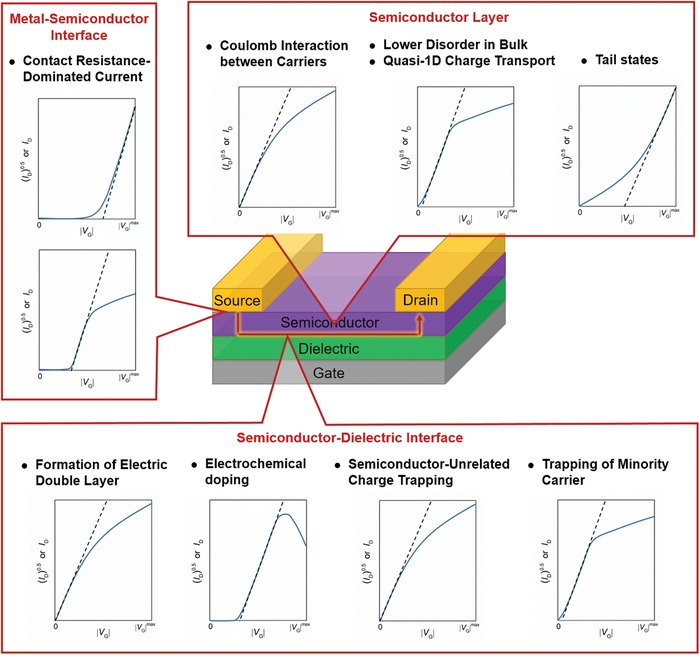
Summary of the relationship between the origins of nonidealities and the resulting shapes of transfer characteristics. Dashed lines represent the extraction methods commonly used in mobility assessment from transfer characteristics (blue lines).

In summary, to understand the nonideal behaviors of OFETs, we described the basic concepts and assumptions of the classic model of OFETs first, and then discussed and analyzed the origins of the nonideal current–voltage characteristics in device physics. The relationship between origins and the shapes of nonidealities is summarized in **Figure** **17**. Lastly a number of solutions to the issues of nonidealities were suggested. As the mobilities of organic semiconductors are now approaching (or even have reached) the requirements for some demanding applications (such as current‐driven flexible OLED displays and radio frequency devices), accurate mobility assessment is critically important for truly reflecting the electrical properties of a material in a device and thus truly guiding the future research fields as well as applications.

To achieve ideal OFETs, in addition to reducing energetic disorder for improving charge transfer, couple of recommendations can be concluded as follows:

i) Improving the metal–semiconductor interface toward ohmic contact via aligning the energy levels of materials, doping, inserting hole/electron transport layer, controlling the thickness of dielectrics, modifying metal with SAMs or polar molecules, and so on.
ii) Choosing a suitable dielectric material (e.g., polymer dielectrics without active functional and polar groups) and avoiding charge injection from semiconductor into dielectric layer and charge trapping at the interface.
iii) Improving the homogeneity and order degree of the semiconductor layer for avoiding large tail states (broad subthreshold region) and different disorder degree between the semiconductor–dielectric interface and the bulk of semiconductor layer.

If the device still exhibits nonideal behavior even after careful device optimization, it is recommended to characterize and report the electrical properties by using Hall mobility and four‐probe measurement, or even to extract the parameters by using a suitable revised model/equation rather than those for conventional FETs. Moreover, the following data should be included in the papers: transfer and output characteristics, mobility versus gate voltage, square root of drain current versus gate voltage for saturation regime, and drain current versus gate voltage for linear regime.

Despite the nonideality puzzles us sometimes, we should emphasize that this issue does not question the fact that there is now a broad range of organic semiconductors with a performance beyond the amorphous silicon. A number of the recently reported high‐mobility materials show ideal transfer characteristics,^62^, ^131^, ^138^, ^139^, ^140^, ^141^ showing the reliability for potential applications in future organic electronics. Progress of high‐performance organic semiconductors and device optimizations is still encouraging. In addition to high mobility, in terms of achieving the utilization of OFETs in a variety of emerging modern applications in flexible, stretchable, transparent, and ultrathin large‐area microelectronics, it should be noted that long‐term operational stability, reliable and predictable electrical behaviors, size minimization, and ohmic contact are equally important.

## Conflict of Interest

The authors declare no conflict of interest.
